# Migraine and cardiovascular disease: A two-sample Mendelian randomization study

**DOI:** 10.1097/MD.0000000000042124

**Published:** 2025-04-18

**Authors:** Shuo Li, Yan Lu, Mengmeng Wang, Rui Li, Hongxiang Gao, Rui Liu, Jiahui He, Dianhui Yang

**Affiliations:** a Shandong University of Traditional Chinese Medicine, Jinan, China; b Affiliated Hospital of Shandong University of Traditional Chinese Medicine, Jinan, China.

**Keywords:** cardiovascular disease, Mendelian randomization, migraine

## Abstract

This study investigates the causal association between migraine and cardiovascular disease from a genetic perspective based on the Mendelian randomization analysis (MR) method. Single nucleotide polymorphism (SNPs) loci were used as genetic instrumental variables (IVs) to analyze potential causal relationships between exposure and outcome factors. The causal association between migraine and cardiovascular disease in terms of prevalence was assessed using inverse variance weighted as the primary MR method. Migraine is negatively associated with coronary artery disease (CAD) when migraine is the exposure and CAD is the outcome (OR = 0.0226, 95% CI = 0.0019–0.2639, *P* = .0024). CAD was negatively associated with migraine when CAD was the exposure and migraine was the outcome (OR = 0.9950, 95% CI = 0.9922–0.9978). No causal association between other cardiovascular diseases and migraine was found. This study demonstrates a negative causal relationship between CAD and migraine.

## 1. Introduction

Migraine is a common and highly disabling neurovascular disorder, often presents as a unilateral, throbbing headache exacerbated by physical activity, accompanied by photophobia and phonophobia as well as gastrointestinal symptoms such as nausea and vomiting.^[1]^ Epidemiological studies show a global age-standardized prevalence rate of 14.4%, which is as high as 18.9% in females compared to 9.8% in males, with the incidence rate increasing yearly this year.^[2]^ According to the Global Burden of Disease 2019 (GBD2019) study, migraine is the second leading cause of disability, as measured by disease-induced disability life-years lost (disability life-years lost refers to all healthy life-years lost from the onset of illness [injury] to death [or recovery]).^[3]^ Cardiovascular disease is a group of diseases involving the heart and blood vessels, including IS, CAD, AF, hypertension, etc, which is characterized by high morbidity, high mortality and high disability, and is a major public health problem that poses a serious threat to human health at present.^[4]^ Some studies have shown that migraine and cardiovascular disease are more likely to occur together than by chance,^[5]^ Given that the evidence for associations between the two is based mostly on observational studies with inherent limitations in accounting for confounding and reverse causation bias, the causality of these associations remains undetermined.

Mendelian randomization (MR) is a research method that uses genetic variants that are strongly correlated with exposure factors as instrumental variables (IVs), which can be used to infer causal effects between exposure factors and study outcomes. Alleles are independently segregated during reproduction and randomly combined and passed on to offspring with equal probability during crossbreeding, so it is generally accepted that genetic variation is randomly distributed at conception^[6]^ MR studies effectively reduce the impact of reverse causality and causal confounding estimates in observational data. In addition, due to the high precision of SNP measurements, measurement errors have less impact on the results.^[7]^

Therefore, this study applied a 2-sample bidirectional MR analysis to assess whether there is a potential causal relationship between selected cardiovascular diseases: ischemic stroke (IS), coronary artery disease (CAD), atrial fibrillation (AF), hypertension, and migraine. Although it has been shown that real-time 3-dimensional speckle-tracking echocardiography enables earlier assessment of CAD after its onset,^[8]^ we hope that the present study will be able to predict the population with a high prevalence of migraine and cardiovascular disease for prevention purposes.

## 2. Method

### 2.1. Ethical approval

Our study was based on publicly available GWAS data and therefore did not require additional ethical approval.

### 2.2. MR study design

**Figure 1.**
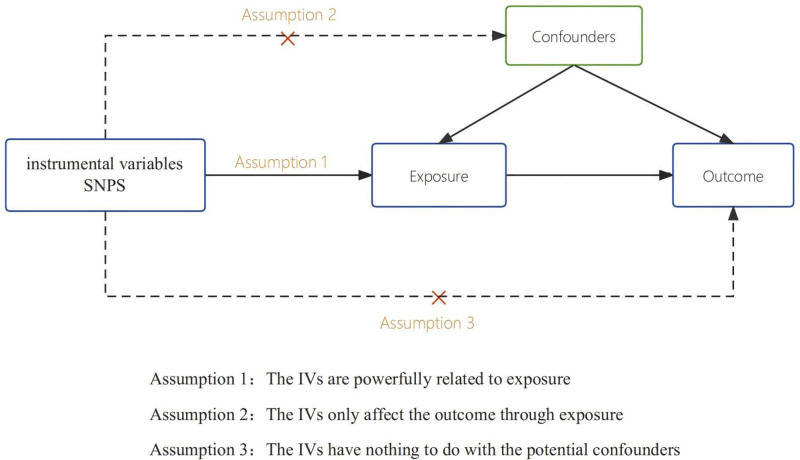
The study design

Figure 1 describes the study design. We used an MR design that utilized a genetic instrumental variable analysis based on summary-level data that instrumented for Single nucleotide polymorphism (SNPs) as risk factors. To ensure the validity of causal estimation in MR studies, 3 key assumptions must be met: Genetic variation is strongly associated with exposure; Genetic variation was not associated with any potential confounders of the exposure-outcome association; Variants don’t have an independent effect on the outcome, except for the association with exposure. To fulfill the above assumptions, we developed the following inclusion criteria: SNPs included in the study were highly correlated with the significance threshold (*P*＜5 × 10^−8^） exposure genome-wide and all included SNPs had to be in linkage equilibrium (*r*^*2*^**＜**0.01). We use the F-statistic to test the significance of the results of the regression analysis; the larger the F-statistic, the more significant the regression proves to be. The F-statistic is calculated as follows: F = R^2^ × (N − k − 1）/[(1 − R^2^）×k],where N is the GWAS sample size for cardiovascular disease, k is the number of SNPs, and R^2^ is the proportion of cardiovascular disease explained by each SNP.

### 2.3. Data sources

GWAS summary data used in this study were obtained from the Ieu Open GWAS database. The sample sizes of the GWAS data for migraine, IS, CAD, AF, and hypertension were 484598, 484121, 547261, 1030836, and 484598 respectively, and 9587836, 24174314, 7934254, 33519037, and 9587836 SNPs. For detailed information on all GWAS data, see Appendix 1, Supplemental Digital Content, http://links.lww.com/MD/O704. To avoid possible bias from race-related confounders, all samples were from European populations.

### 2.4. MR analysis

In order to determine whether there is a causal relationship between autoimmune system disorders and migraine, we focused on MR analysis using the inverse variance weighting (IVW) approach. The IVW estimate is a measure of the combined overall effect size of the Wald ratio estimates for individual SNPs. IVW was used to integrate the individual effect estimates of multiple SNPs if multiple IVs were included and the 3 hypotheses of the MR analysis were met, when the IVW method is very valid and reliable.^[9]^ We also used 4 other methods for MR analysis (the weighted median, MR-Egger, the simple mode, and the weighted mode）. The weighted median method (WME) can estimate the heterogeneity of causal effects when at least 50% of the SNPs are valid IVs.^[10]^ When there is polytropy in IV, it can be estimated using MR-Egger regression.^[11]^ The sample mode and the weighted mode aim to obtain a single IV causal effect estimate from multiple IVs, and they have the advantage of smaller bias and lower probability of Type I error.^[12,13]^ When only one instrumental variable was included in the MR model, the Wald Ratio method was used for the estimation of the causal effect of the exposure variable and disease outcome.^[14]^ We used the TwosampleMR package in the R software for the entire data analysis and assessed the strength of the association using the ratio of ratios (OR), where exposure was a risk factor for the outcome when the OR value was > 1, a protective factor for the outcome when the OR value was < 1, and had no effect on the outcome when the OR value = 0.

### 2.5. Sensitivity analysis

Sensitivity analyses are conducted to measure the feasibility and stability of conclusions. Horizontal pleiotropy was then judged by the intercept term of the MR-Egger regression, with *P* < .05 indicating the presence of horizontal pleiotropy. To further investigate the possibility of horizontal pleiotropy, we used MR-PRESSO to explore horizontal pleiotropy, which involves eliminating outliers in the data.^[15]^ Heterogeneity was assessed using Cochran Q statistic, with *P* < .05 indicating the presence of heterogeneity.^[16]^ Sensitivity analyses using the “leave-one-out” method (i.e., removing each SNP in turn and calculating the combined effect of the remaining SNPs) were performed to evaluate whether individual SNPs had an effect on the relationship between exposure and outcome. Results were visualized using funnel plots and forest plots. All MR analyses were performed using the “TwoSampleMR” package in R version 4.3.1.

## 3. Result

### 3.1. Causal relationship between migraine and cardiovascular disease

**Figure 2.**
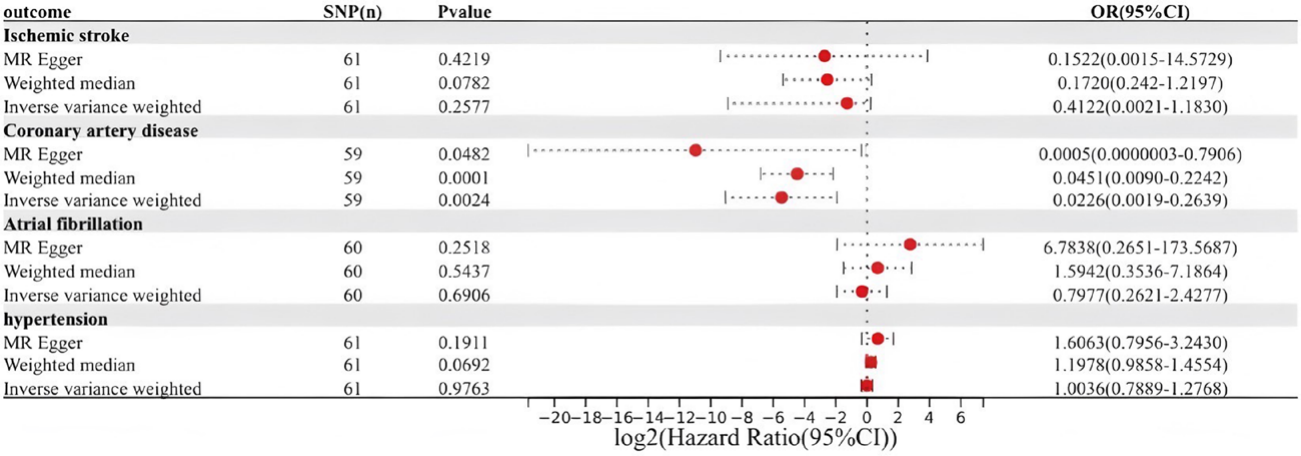
Causal effect of migraine on cardiovascular disease. CI = confidence interval.

**Figure 3.**
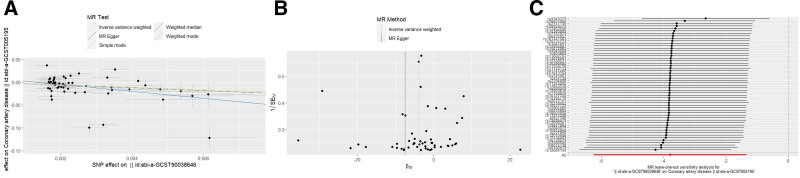
Effect of migraine on CAD. (A) Scatterplot with the slope of each line indicating the causal relationship of each method. (B) Funnel plot. (C) Leave-one-out analysis. CAD = coronary artery disease.

During the course of our study, we found that the number of SNPs associated with migraine was insufficient when applying a statistical threshold of *P* < 5 × 10^−8^. To address this limitation, the correlation threshold was adjusted to use a more relaxed significance criterion of *P* < 5 × 10^−6^. IVW as the primary analysis showed that migraine was negatively associated with CAD (OR = 0.0226, 95% CI = 0.0019–0.2639, *P* = .0024). The other 2 MR analysis methods (MR-Egger: OR = 0.0005, 95% CI = 0.0000003–0.7906, *P* = .0482; Weighted median: OR = 0.0451, CI = 0.0090–0.2242, *P* = .0001) were in agreement with the IVW directions and results (Fig. 2). In the analysis of heterogeneity tests, Cochran Q test using IVW and MR-Egger methods found no evidence of heterogeneity. No evidence of directed pleiotropy using MR-Egger intercept test results. The leave-one-out method of analysis suggests that the exclusion of individual SNPs does not lead to substantial differences in the estimates of the combined effects between the remaining SNPs and the overall results. This result emphasizes the robustness of the MR estimation results, and the remaining analyses also demonstrate the robustness of the MR effect estimates (Fig. 3); (Table 1). However, the IVW approach did not demonstrate any statistically significant relationship between migraine and other cardiovascular diseases.

**Table 1:** Heterogeneity and polytropy tests.

Exposure	Outcome	Heterogeneity test						Pleiotropy test		
		MR-Egger			Inverse variance weighted			MR-Egger		
		Q	Q_df	*P*-value	Q	Q_df	*P*-value	Intercept	SE	*P*-value
Migraine	Ischemic stroke	108.426	59	9.38E-05	108.8061	60	1.18E-04	0.002565389	0.005640592	.6509161
Migraine	Coronary artery disease	377.1749	52	7.18E-51	385.4351	53	5.43E-52	0.00967901	0.00907001	.2908361
Migraine	Atrial fibrillation	88.3491	58	0.006266865	91.23504	59	0.004502962	−0.005541779	0.004026176	.1739771
Migraine	hypertension	303.8233	59	1.19E-34	313.8496	60	4.62E-36	−0.001212174	0.000868718	.1681377

MR = Mendelian randomization, Q = heterogeneity statistic, Q_df = degrees of freedom, SE = standard error.

### 3.2. Causal effect of cardiovascular disease on migraine

**Figure 4.**
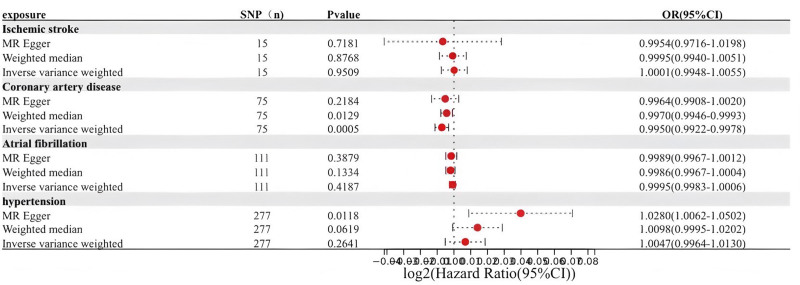
Causal effect of cardiovascular disease on migraine. CI = confidence interval.

**Figure 5.**
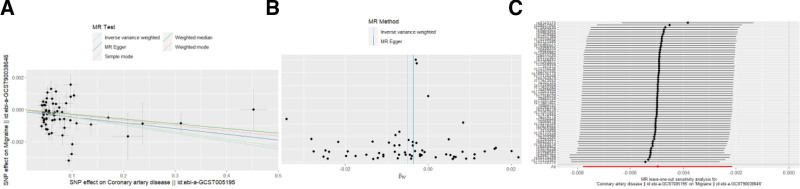
Effect of CAD on migraine. (A) Scatterplot with the slope of each line indicating the causal relationship of each method. (B) Funnel plot. (C) Leave-one-out analysis. CAD = coronary artery disease.

IVW as the primary analysis showed that CAD was negatively associated with migraine headaches (OR = 0.9950, 95% CI = 0.9922–0.9978), Weighted median analysis supports this view (OR = 0.9970, 95% CI = 0.9946–0.9993, *P* = .0129); (Fig. 4）. Cochran Q test using IVW and MR-Egger methods found no evidence of heterogeneity. No evidence of directed pleiotropy using MR-Egger intercept test results. The leave-one-out method of analysis suggests that the exclusion of individual SNPs does not lead to substantial differences in the estimates of the combined effects between the remaining SNPs and the overall results. This result emphasizes the robustness of the MR estimation results, and the remaining analyses also demonstrate the robustness of the MR effect estimates (Fig. 5); (Table 2). Similarly, the IVW approach did not demonstrate any statistically significant relationship between other cardiovascular diseases and migraine.

**Table 2:** Heterogeneity and polytropy tests.

Exposure	Outcome	Heterogeneity test						Pleiotropy test		
		MR-Egger			Inverse variance weighted			MR-Egger		
		Q	Q_df	*P*-value	Q	Q_df	*P*-value	Intercept	SE	*P*-value
Ischemic stroke	Migraine	29.22059	12	.003653357	29.59755	13	.005380267	0.000289397	0.000735527	.7008846
Coronary artery disease	Migraine	240.2589	60	2.04E-23	241.4732	61	2.61E-23	0.000119973	0.000217858	.5838901
Atrial fibrillation	Migraine	152.2356	109	.003959649	152.6267	110	.004491091	4.75E-05	8.98E-05	.5977478
hypertension	Migraine	496.4951	268	7.31E-16	506.098	269	1.04E-16	−0.000198129	8.70E-05	.02359074

MR = Mendelian randomization, Q = heterogeneity statistic, Q_df = degrees of freedom, SE = standard error.

## 4. Discuss

Several studies have shown an independent association between migraine and IS,^[17, 18]^ and it has been hypothesized that the mechanism may be related to cortical spreading depression, endothelial dysfunction, hypercoagulability, and genetics.^[17, 19–21]^ A prospective community-based atherosclerosis risk study found that migraine was associated with an increased risk of developing AF by following participants with a history of migraine and AF.^[22]^ Some studies have elucidated the common pathogenesis between migraine and hypertension in terms of endothelial dysfunction and impaired nitric oxide, and the angiotensin system, suggesting that there is a strong link between the two.^[23, 24]^ The present study showed no causal relationship between migraine and IS, AF, and hypertension from a genetic point of view, suggesting that migraine is not a risk factor for these diseases. Our study also found that CAD and migraine were protective factors for each other, with migraine being able to greatly reduce the risk of developing CAD, and CAD reducing the incidence of migraine to some extent. This also suggests that we should be concerned about whether medications used to prevent and treat migraine lead to an increased risk of CAD. In recent years, with the exploration of its pathogenesis, calcitonin gene-related peptide (CGRP) has been found to play an important role in the pathogenesis of migraine headache.^[25]^ CGRP analogs (monoclonal antibodies and small molecule receptor antagonists) are commonly used as prophylaxis for migraine headaches. The monoclonal antibodies and small molecule receptor antagonists that have been approved by the U.S. Food and Drug Administration are erenumab, fremanezumab, galcanezumab, eptinezumab, rimegepant, atogepant, ubrogepant, and zavegepant respectively. It is reassuring that several studies have shown a favorable safety profile for this class of drugs. CGRP monoclonal antibodies are injectable drugs, therefore, their adverse reactions are mainly injection site-related discomfort, including pain, rash and hardness. The common adverse effects of zavegepant are taste disturbance and nasal discomfort also explained by the specificity of its nasal spray. In the small molecule receptor antagonist class of drugs, the most common adverse effect was constipation, and none of them mentioned serious adverse events such as cardiovascular.^[26–28]^ However, most of the clinical trials of CGRP analogs have a short lead time, as well as fewer real-world studies, and the risks and safety of long-term use of CGRP analogs are still debatable.

The present study is diametrically opposed to the observations of many previous epidemiologic studies. One possible reason for this is that migraineurs often have concomitant abnormalities in cardiovascular risk factors, such as hypertension, hyperglycemia, and abnormal lipids, and face an increased risk of cardiovascular events even after adjusting for these factors.^[29]^ There have been studies proposing that blood pressure variability has an effect on carotid atherosclerosis.^[30]^ Therefore, the association between migraine and cardiovascular disease found in observational studies cannot be viewed as causal. A key strength of this study relative to observational epidemiology is that MR methods are less subject to confounding and reverse causation.

There are several limitations to our study: We used exposure and outcome genomic data exclusively from the European cohort. Therefore, the conclusions drawn from this study may have limitations in relation to populations with high ethnic and genetic diversity and should subsequently be validated in other populations. There are also instances where sample overlap may bias estimates from 2-sample MR studies. The scope of this study was limited to exploring causal interactions between 4 cardiovascular diseases and migraine that have been the subject of previous calls for observational studies. Unfortunately, it was not possible to cover all cardiovascular diseases in this study. The association between cardiovascular disease and migraine is influenced by more than just genetic factors. Although our PhenoScanner GWAS dataset excludes confounding factors associated with IV, we are still unable to completely eliminate the influence of other factors. Although we performed a comprehensive sensitivity analysis and did not detect any pleiotropy, we cannot exclude that potential horizontal pleiotropy may exist, which could lead to biased results in MR studies.

## 5. Conclusion

Our study suggests a negative causal relationship between CAD and migraine. This finding should be fully considered in the development of public health policies and prevention strategies for migraine and CAD. There is no evidence to support a causal relationship between other cardiovascular diseases and migraine.

## Author contributions

**Conceptualization:** Mengmeng Wang, Rui Li, Hongxiang Gao, Rui Liu, Jiahui He.

**Data curation:** Yan Lu.

**Funding acquisition:** Dianhui Yang.

**Writing – original draft:** Shuo Li.

**Writing – review & editing:** Shuo Li, Dianhui Yang.

## Supplementary Material


