# Supplementary material for: Migraine and cardiovascular disease: A two-sample Mendelian randomization study
Source: Medicine (Baltimore). 2025 Apr 18;104(16):e42124. doi: 10.1097/MD.0000000000042124 (PMC12014051; doi:10.1097/MD.0000000000042124)
Supplement: Supplementary file 1 [file medi-104-e42124-s001.docx]

| Phenotypes | GWAS ID | Cases | Controls | Sample size | Population | Study/Consortium | PubMed ID | Year |
| --- | --- | --- | --- | --- | --- | --- | --- | --- |
|  |  |  |  |  |  |  |  |  |
| Migraine | [ebi-a-GCST90038646](https://gwas.mrcieu.ac.uk/datasets/ebi-a-GCST90038646/) | 13971 | 470627 | 484598 | NA | NA | 33959723 | 2021 |
| Ischemic stroke | [ebi-a-GCST90018864](https://gwas.mrcieu.ac.uk/datasets/ebi-a-GCST90018864/) | 11929 | 472,192 | 484,121 | European | NA | 34594039 | 2021 |
| Coronary artery disease | [ebi-a-GCST005195](https://gwas.mrcieu.ac.uk/datasets/ebi-a-GCST005195/) | 122733 | 424,528 | 547261 | NA | NA | 29212778 | 2017 |
| hypertension | [ebi-a-GCST90038604](https://gwas.mrcieu.ac.uk/datasets/ebi-a-GCST90038604/) | 129909 | 354689 | 484598 | NA | NA | 33959723 | 2021 |
| myocardial infarction | ebi-a-GCST90038610 | 11,081 | 473,517 | 484598 | NA | NA | 33959723 | 2021 |
